# Supplementary material for: Transient local adaptation and source–sink dynamics in experimental populations experiencing spatially heterogeneous environments
Source: Proc Biol Sci. 2019 Jun 26;286(1905):20190738. doi: 10.1098/rspb.2019.0738 (PMC6599998; doi:10.1098/rspb.2019.0738)
Supplement: Tables S1 - S2 and Fig. S1 [file rspb20190738supp1.pdf]

SUPPLEMENTARY INFORMATION

Bisschop, K., Mortier, F., Etienne, R. S., Bonte, D. (2019) Transient local adaptation and source-sink dynamics in experimental populations experiencing spatially heterogeneous environments, *Proceedings of the Royal Society B,* doi: 10.1098/rspb

Table S1: **Model selection for the fecundity tests**. Overview of the best models based on the lowest AICc with an AICc weight of at least 0.100. The total number of models was five in the control population and nineteen for experimental populations. Resource in the model is the homogeneous or heterogeneous environment and plant species where the mite came from.

	Model	df	LogLik	AICc	Δ AICc	AICc weight
**Ancestral versus novel host plants<br>maximal model: performance ~ time * experiment/control + (1|block/island)**						
Bean	**Time * Experiment/Control**	13	-4797.299	9620.9	0.00	0.622
	Time	8	-4803.254	9622.6	1.72	0.264
	Time + Experiment/Control	9	-4803.081	9624.3	3.40	0.114
Cucumber	**Experiment/Control**	5	-4079.505	8169.1	0.00	0.668
	No fixed variables	4	-4081.728	8171.5	2.43	0.199
	**Time**	9	-1466.831	2951.8	0.00	0.642
Pepper	Time + Experiment/Control	10	-1466.640	2953.5	1.66	0.281
**Interspecific competition and time<br>maximal model: performance ~ competition * resource * time + (1|block/island)**						
Bean	**Competition + Time**	9	-4108.458	8235.1	0.00	0.755
	Competition + Time + Resource	11	-4108.178	8238.6	3.53	0.129
Cucumber	**Competition**	5	-3482.708	6975.5	0.00	0.240
	No fixed variables	4	-3483.866	6975.8	0.29	0.207
	Competition * Resource	9	-3479.502	6977.2	1.72	0.101
	Competition + Time	9	-3479.509	6977.2	1.74	0.101
Pepper	**Time**	8	-1291.881	2599.9	0.00	0.467
	Resource + Time	10	-1290.537	2601.3	1.40	0.232
	Competition + Time	9	-1291.831	2601.9	1.94	0.177

Table S2: **Chi-square statistics for the maximal models before model selection**. The results for the Wald Chi-square tests are presented for the maximal models. The fist column with bean, cucumber, and pepper indicates the plant species where fecundity was measured on.

	Independent variables	Chisq	Df	Pr(>Chisq)	
**Ancestral versus novel host plants**					
Bean	Time	88.0081	4	<2.00E-16	***
	Experiment/Control	0.3495	1	0.55441	
	Time : Experiment/Control	11.6228	4	0.02039	*
Cucumber	Time	4.244	4	0.37399	
	Experiment/Control	5.6337	1	0.01762	*
	Time : Experiment/Control	2.4356	4	0.65621	
Pepper	Time	81.5728	4	<2e-16	***
	Experiment/Control	0.2015	1	0.6535	
	Time : Experiment/Control	5.7604	4	0.2178	
**Interspecific competition and time**					
Bean	Resource	0.5778	2	0.749085	
	Competition	8.7494	1	0.003097	**
	Time	73.7348	4	3.69E-15	***
	Resource : Competition	0.6358	2	0.727665	
	Resource : Time	7.8797	8	0.445308	
	Competition : Time	1.1322	4	0.889135	
	Resource : Competition : Time	8.293	8	0.405389	
Cucumber	Resource	1.6767	2	0.43243	
	Competition	2.8922	1	0.08901	.
	Time	6.0704	4	0.19395	
	Resource : Competition	4.5489	2	0.10285	
	Resource : Time	3.0794	8	0.92928	
	Competition : Time	6.0623	4	0.19454	
	Resource : Competition : Time	19.2584	8	0.01354	*
Pepper	Resource	3.3926	2	0.1834	
	Competition	0.1135	1	0.7362	
	Time	41.805	4	1.83E-08	***
	Resource : Competition	0	2	1	
	Resource : Time	6.4407	8	0.598	
	Competition : Time	2.249	4	0.6901	
	Resource : Competition : Time	7.4229	8	0.4918	
**Comparison between mites taken from cucumber and pepper in heterogeneous islands**					
Bean	Host plant	0.0586	1	0.8087	
	Time	45.3405	4	3.38E-09	***
	Host plant : Time	3.9073	4	0.4187	
Cucumber	Host plant	1.1013	1	0.294	
	Time	4.74	4	0.315	
	Host plant : Time	1.5975	4	0.8092	
Pepper	Host plant	0.1153	1	0.73422	
	Time	63.2495	4	6.01E-13	***
	Host plant : Time	10.2238	4	0.03682	*

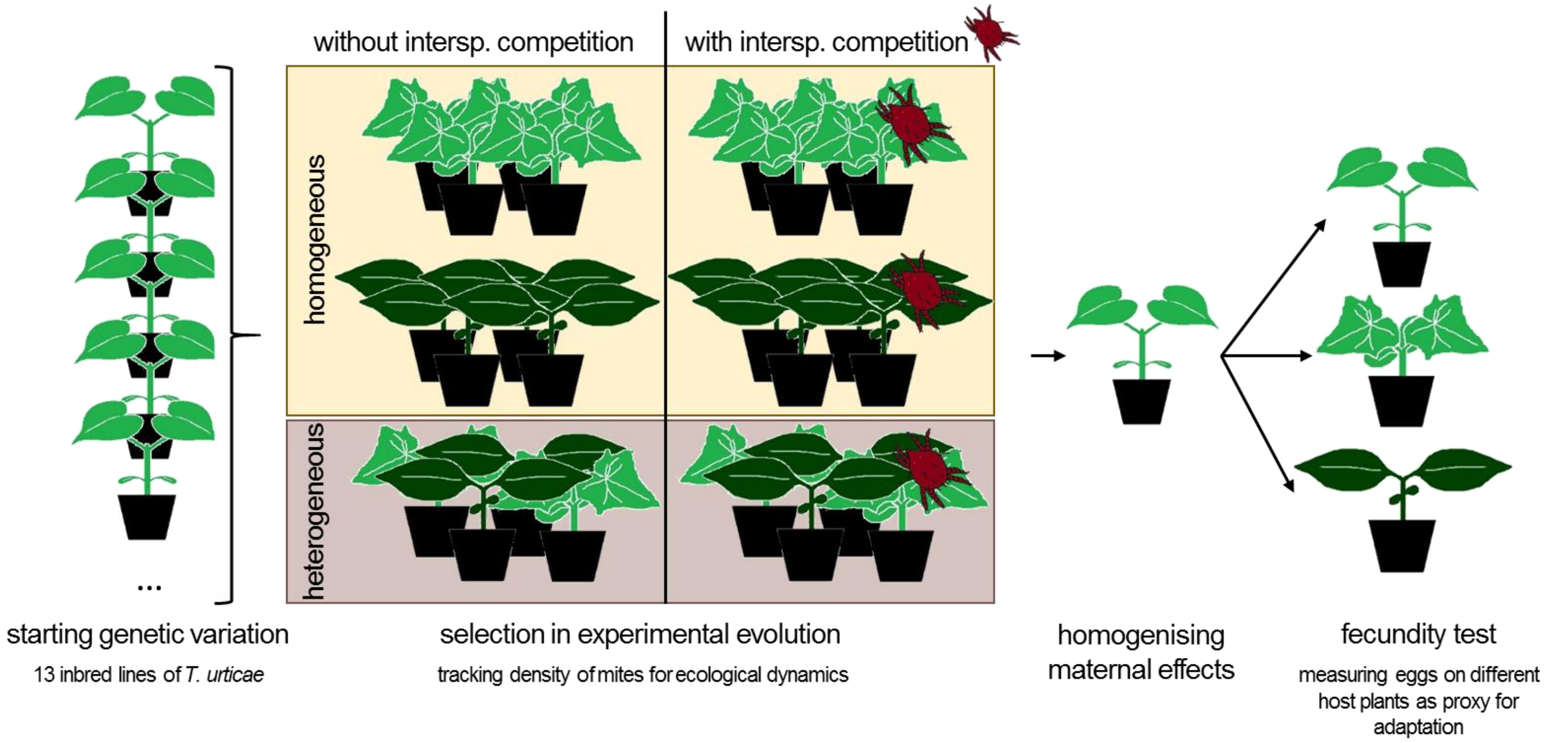

Fig. S1. The experimental set-up. Adult females from 13 inbred lines of *T. urticae* were equally divided over the different treatments to create the same starting genetic variation. The treatments were homogeneous islands with cucumber or pepper (yellow box), or heterogeneous with the combination of the two (brown box). Half of the islands was without interspecific competition, while the other half was with. The density of the populations of mites was tracked for ecological dynamics and fecundity tests were performed on the novel and initial host plants after two generations on the initial host plant for homogenising maternal effects.
